# Optical Coherence Tomography Angiography Findings after Acute Intraocular Pressure Elevation in Patients with Diabetes Mellitus versus Healthy Subjects

**DOI:** 10.18502/jovr.v17i3.11573

**Published:** 2022-08-15

**Authors:** Maryam Ashraf Khorasani, Giancarlo A Garcia, Pasha Anvari, Abbas Habibi, Shahriar Ghasemizadeh, Khalil Ghasemi Falavarjani

**Affiliations:** ^1^Eye Research Center, The Five Senses Institute, Iran University of Medical Sciences, Tehran, Iran; ^2^Byers Eye Institute at Stanford University, Palo Alto, CA, USA; ^3^Stem Cell and Regenerative Medicine Research Center, Iran University of Medical Sciences, Tehran, Iran

**Keywords:** Diabetic Retinopathy, Glaucoma, Intraocular Pressure, Macula, Ocular Blood Flow, Ocular Perfusion, Optic Nerve, Optical Coherence Tomography Angiography, Retinal Imaging; Vessel Density

## Abstract

**Purpose:**

To assess the changes in optic nerve head and macular microvascular networks after acute intraocular pressure (IOP) rise in healthy eyes versus the eyes of diabetic patients.

**Methods:**

In this prospective, interventional, comparative study, 24 eyes of 24 adults including 12 eyes of healthy nondiabetic subjects and 12 eyes with mild or moderate non-proliferative diabetic retinopathy (NPDR) were enrolled. IOP elevation was induced by a suction cup attached to the conjunctiva. IOP and optical coherence tomography angiographic (OCTA) images of the optic disc and macula were obtained before and immediately after the IOP rise.

**Results:**

Baseline and post-suction IOPs were not significantly different between the two groups (all *P* > 0.05). The mean IOP elevation was 13.93 ± 3.41 mmHg among all eyes and was statistically significant as compared to the baseline in both groups (both *P* < 0.05). After IOP elevation, healthy eyes demonstrated a reduction in the vessel density in the whole image deep and superficial capillary plexuses and parafoveal deep capillary plexus (DCP) (all *P* < 0.05). In diabetic retinopathy, foveal vessel density at DCP decreased significantly following IOP rise (*P* = 0.003). In both groups, inside disc vessel density decreased significantly after IOP rise (both *P* < 0.05), however, no significant change was observed in peripapillary vessel density (both *P* > 0.05).

**Conclusion:**

Acute rise of IOP may induce different levels of microvascular changes in healthy and diabetic eyes. Optic disc microvasculature originating from the posterior ciliary artery may be more susceptible to IOP elevation than that of retinal microvasculature.

##  INTRODUCTION

Glaucoma is a major cause of irreversible vision loss and one of the leading causes of blindness worldwide.^[1,2]^ Several studies have demonstrated that structural changes that occur in the retina and optic nerve in glaucoma are preceded by functional disorders.^[3,4,5,6,7,8,9,10]^

The exact mechanism by which glaucoma leads to structural changes has not yet been well understood. Both mechanical and vascular theories have been proposed to describe the pathogenesis of glaucoma-induced structural changes, though neither of these theories alone is sufficient to fully explain the disease mechanism. In both theories, however, increased intraocular pressure (IOP) plays an important role.^[11,12]^

The mechanical theory proposes that increased IOP compresses intraocular structures and attenuates axoplasmic flow leading to glaucomatous changes. In the vascular theory, it is believed that impaired blood supply leads to structural damages.^[12,13,14]^ The latter theory is supported by findings that link systemic hypotension to increased risk of glaucomatous optic neuropathy.

Autoregulation is a mechanism that, despite the changes in ocular perfusion pressure (OPP), maintains a relatively constant level of ocular blood flow (OBF) and provides sufficient oxygen and nutrients for the cells.^[15]^ OPP is expressed as the difference between the arterial blood pressure and the IOP. Accordingly, a reduction in blood pressure or an increase in IOP leads to a reduction of OPP. In normal eyes, autoregulation compensates for these changes, and blood flow to the eye remains constant. If, however, autoregulation is impaired, or IOP surpasses a threshold and the eye is unable to compensate, OBF becomes unstable; in this context, small changes in IOP lead to changes in OBF that can induce glaucomatous changes.^[16,17,18,19]^

Dysfunctional ocular autoregulation that plays an important role in the development and progression of glaucoma occurs in diabetic patients as well.^[18,19]^ The pathophysiology of diabetes mellitus also involves dysfunctional vascular regulation, where the disease is believed to share an association with glaucoma, although a definitive link has not been established.^[18,19]^

Various modalities have been utilized to asses OBF changes after acute IOP rise, including Doppler sonography,^[20,21]^ laser interferometry,^[20,22]^ and laser Doppler flowmetry.^[21,22]^ However, these methods are limited by their invasive nature and inability to record all retinal vascular layers simultaneously. Optical coherence tomography angiography (OCTA), on the other hand, is a noninvasive technology that enables the visualization of blood vessels in different layers of the retina and choroid without dye injection.^[23]^ This modality employs consecutive optical coherence tomography (OCT) b-scans captured from the same location, comparing the decorrelation signal between scans to identify blood vessels and map blood flow.^[24]^ Previous studies have shown that OCTA is a reliable imaging modality for the diagnosis of different retinal, choroidal, and optic nerve diseases.^[25,26,27]^

We herein utilized OCTA to assess changes in the optic nerve head, the macular vasculature, as well as retinal thickness following acute IOP rise, and compared these changes in the eyes of healthy versus diabetic subjects. To the best of our knowledge, this is the first study assessing changes in the optic nerve head and macular vasculature as well as the retinal thickness around the optic disc and in the macular area following the acute rise of IOP in normal versus diabetic subjects.

##  METHODS

This prospective interventional comparative study was conducted at the Ophthalmology Clinic of the Rassoul Akram Hospital, Iran University of Medical Sciences. Participants were enrolled between the periods of September and December 2018. The Ethics Committee of the Iran University of Medical Sciences approved the study protocol (IR.IUMS.REC.1398.200). Informed consent was obtained from the patients, and the study was conducted in accordance with the tenets of the Helsinki Declaration.

In this study, nondiabetic healthy subjects with no ocular disorders and patients with mild or moderate non-proliferative diabetic retinopathy (NPDR group) were included. All participants underwent a standard baseline comprehensive ophthalmic examination that included the Snellen best-corrected visual acuity (BCVA) measurements, IOP measurements using a rebound tonometer (Icare ic100, IcareⓇ, Vantaa, Finland), slit-lamp examination, and dilated fundus examination.

All included eyes had to have an IOP of < 22mmHg and spherical equivalent refraction between –6 and +4 diopters. Healthy eyes had a BCVA of 20/20 or better with normal comprehensive ocular examinations. Inclusion criteria for the NPDR group were type 2 diabetics who were taking only oral antidiabetic medications, with clinical evidence of mild or moderate NPDR in both eyes according to the Early Treatment of Diabetic Retinopathy Study (ETDRS) grading criteria.^[28]^

Exclusion criteria were any media opacity (e.g., cataract or vitreous hemorrhage) that might interfere with imaging, eyes with an OCT scan quality of < 7, and a history of any ocular surgery or ocular laser treatment.

### Image Acquisition and Elevation of IOP

OCTA imaging was performed by a RTVue XR Avanti device (Version 2017.1.0.151, Optovue, Inc., Fremont, CA, USA). Baseline OCTA images included macular (3 × 3) and optic disc (4.5 × 4.5) scans in the same session. IOP was recorded using an Icare device at baseline. The procedure for inducing IOP elevation has been described elsewhere.^[29]^ In brief, after achieving topical anesthesia using tetracaine 0.5% ophthalmic solution, the episcleral suction cup was attached to the temporal conjunctiva to apply negative pressure to the globe. Immediately after the application of the suction cup, IOP was recorded again while the suction cup was stable and the imaging was repeated with the same macular and optic disc protocols, with the suction cup in place. A drop of artificial tears was administered before post-suction measurements to avoid dry eye-induced image distortion. A minimum increase of 5 mmHg in IOP was needed to proceed to post-suction image acquisition. If there was slippage of the suction cup, all measurements were postponed to another day.

The parameters extracted included retinal vessel density at fovea and parafovea in the deep capillary plexus (DCP) and at the superficial capillary plexus (SCP) layers. The SCP en face image was automatically segmented with an inner boundary set at the internal limiting membrane (ILM) and an outer boundary set at 9 µm above the inner plexiform layer (IPL). The DCP en face image was segmented with an inner boundary set at 9 µm above the IPL and an outer boundary at 9 µm below the outer plexiform layer (OPL). Peripapillary and inside disc vessel density measurements were automatically recorded from the ILM to the lower border of the nerve fiber layer. From the AngioDisc images, the peripapillary retinal nerve fiber layer (RNFL) thickness as well as the vessel density of the peripapillary and inside disc regions were recorded. “Inside disc vessel density” was defined as the percentage of vessels inside the borders of the optic disc that was automatically determined by the OCTA software. Peripapillary vessel density was calculated for the 750 µm wide ring-shaped area that is located circumferentially around the periphery of the optic disc margins.

**Table 1:** Baseline characteristics in the two groups.


	**Healthy eyes**	**NPDR eyes**	** P -value**
Number of eyes	12	12	
Age (Years)	40.00 ± 11.38	56.17 ± 12.11	0.03
Intraocular pressure (mmHg)	15.28 ± 2.95	17.34 ± 3.29	0.121
Whole image SCP vessel density (%)	47.47 ± 2.29	39.84 ± 5.38	< 0.001
Foveal SCP vessel density (%)	18.22 ± 5.25	11.10 ± 5.09	0.003
Parafoveal SCP vessel density (%)	50.22 ± 2.50	41.97 ± 6.22	0.001
Whole image DCP vessel density (%)	49.50 ± 2.72	45.06 ± 5.02	0.016
Foveal DCP vessel density (%)	32.92 ± 6.53	25.21 ± 8.18	0.018
Parafoveal DCP vessel density (%)	51.32 ± 2.64	47.23 ± 5.16	0.026
Whole image disc vessel density (%)	50.70 ± 1.22	48.46 ± 3.22	0.041
Peripapillary vessel density (%)	53.64 ± 1.27	51.03 ± 4.09	0.055
Inside disc vessel density (%)	49.97 ± 2.68	48.04 ± 3.48	0.142
Peripapillary RNFL thickness (µm)	114.92 ± 12.39	105.08 ± 12.59	0.067
	
	
The data is presented as mean ± SD. SCP, superficial capillary plexus; DCP, deep capillary plexus; RNFL, retinal nerve fiber layer; DM, diabetes mellitus; NPDR, non-proliferative diabetic retinopathy			

**Table 2:** Baseline and post-suction values in healthy eyes.


	**Baseline**	**Post-suction**	***P*** **-value**
IOP (mmHg)	15.28 ± 2.95	28.52 ± 3.33	< 0.001
Whole image SCP vessel density (%)	47.47 ± 2.29	45.86 ± 2.54	0.015
Foveal SCP vessel density	18.22 ± 5.25	17.67 ± 5.29	0.336
Parafoveal SCP vessel density (%)	50.22 ± 2.50	48.75 ± 2.54	0.050
Whole image DCP vessel density (%)	49.50 ± 2.72	47.22 ± 3.78	0.005
Foveal DCP vessel density	32.92 ± 6.53	33.06 ± 6.49	0.745
Parafoveal DCP vessel density (%)	51.32 ± 2.64	49.46 ± 3.70	0.010
Whole image disc density (%)	50.70 ± 1.22	49.67 ± 2.43	0.081
Peripapillary vessel density (%)	53.64 ± 1.27	53.12 ± 2.63	0.365
Inside disc vessel density (%)	49.97 ± 2.68	47.46 ± 4.15	0.017
Peripapillary RNFL thickness (µm)	114.92 ± 12.39	114.83 ± 11.91	0.862
	
	
IOP, intraocular pressure; SCP, superficial capillary plexus; DCP, deep capillary plexus; RNFL, retinal nerve fiber layer			

**Table 3:** Baseline and post-suction measurements in eyes with nonproliferative diabetic retinopathy.


	**Baseline**	**Post-vacuum**	***P*** **-value**
IOP (mmHg)	17.34 ± 3.29	31.96 ± 5.30	< 0.001
Whole image SCP vessel density (%)	39.84 ± 5.38	38.64 ± 5.95	0.133
Foveal SCP vessel density	11.10 ± 5.09	10.52 ± 5.37	0.134
Parafoveal SCP vessel density (%)	41.97 ± 6.22	40.85 ± 6.79	0.179
Whole image DCP vessel density (%)	45.06 ± 5.02	43.87 ± 3.62	0.226
Foveal DCP vessel density	25.21 ± 8.18	24.25 ± 7.93	0.003
Parafoveal DCP vessel density (%)	47.23 ± 5.16	46.28 ± 3.64	0.313
Whole image disc density (%)	48.46 ± 3.22	47.74 ± 2.66	0.233
Peripapillary vessel density (%)	51.03 ± 4.09	51.12 ± 3.01	0.901
Inside disc vessel density (%)	48.04 ± 3.48	44.35 ± 3.77	0.007
Peripapillary RNFL thickness (µm)	105.08 ± 12.59	103.33 ± 12.31	0.005
	
	
IOP, intraocular pressure; SCP, superficial capillary plexus; DCP, deep capillary plexus; RNFL, retinal nerve fiber layer			

**Table 4:** Comparison of the measurement changes between healthy eyes and eyes with nonproliferative diabetic retinopathy after acute rise of intraocular pressure after adjustment for age.


	**Group 1 (healthy eyes)**	**Group 2 (NPDR)**	***P*** -value ${}^{*}$
IOP (mmHg)	13.24 ± 3.97	14.62 ± 2.73	0.243
Whole image SCP vessel density (%)	–1.62 ± 1.93	–1.20 ± 2.56	0.642
Foveal SCP vessel density	–0.56 ± 1.92	–0.57 ± 1.23	0.774
Parafoveal SCP vessel density (%)	–1.47 ± 2.31	–1.12 ± 2.71	0.767
Whole image DCP vessel density (%)	–2.27 ± 2.22	–1.20 ± 3.24	0.332
Foveal DCP vessel density	0.14 ± 1.47	-0.96 ± 0.85	0.155
Parafoveal DCP vessel density (%)	–1.87 ± 2.08	–0.95 ± 3.11	0.966
Whole image disc density (%)	–1.03 ± 1.86	–0.72 ± 1.96	0.202
Peripapillary vessel density (%)	0.52 ± 1.92	0.08 ± 2.27	0.143
Inside disc vessel density (%)	–2.51 ± 3.09	–3.69 ± 3.90	0.298
Peripapillary RNFL thickness (µm)	–0.08 ± 1.62	–1.75 ± 1.71	0.011
	
	
SCP, superficial capillary plexus; DCP, deep capillary plexus; RNFL, retinal nerve fiber layer; DM, diabetes mellitus; NPDR, non-proliferative diabetic retinopathy ${}^{*}$ *P*-value adjusted for age and baseline vessel density measurements using general linear model.			

### Statistical Analysis

The SPSS software version 18.0 (SPSS, Inc., Chicago, IL, USA) and Medcalc software version 19 (MedcalcSoftware, Mariakerke, Belgium) were utilized for statistical analysis. *P*-values < 0.05 were considered statistically significant. All continuous variables were expressed as mean ± standard deviation (SD). Paired samples *t*-test was utilized to compare variables before and after the IOP rise. The general linear model was used to adjust for differences in age and baseline vessel density measurements.

##  RESULTS

In this study, 24 eyes (12 healthy eyes and 12 eyes with NPDR) of 24 adult patients were included. Baseline characteristics are shown in Table 1. The mean age was 40.00 ± 11.38 and 56.17 ± 12.11 years in healthy and NPDR eyes, respectively (*P* = 0.03). The baseline IOP was 15.28 ± 2.95 mmHg and 17.34 ± 3.29 mmHg in healthy and NPDR eyes, respectively (*P* = 0.121).

At baseline, the retinal vessel density for whole image, foveal and parafoveal SCP and DCP was significantly lower in the NPDR group as compared to the healthy eyes (all *P* < 0.05). While vascular density of the whole optic nerve head was significantly lower in eyes with NPDR as compared to the normal eyes (*P* = 0.041), there were no statistically significant differences in the peripapillary and inside disc vessel densitie between the two groups.

The mean increase in IOP after scleral suction was 13.24 mmHg in healthy eyes (range, 6.60–19.40) and 14.62 mmHg in NPDR eyes (range, 11.0–19.90), corresponding to a mean of 13.93 ± 3.41 mmHg among all eyes. A post-suction increase of > 9 mmHg was experienced by 90% of the healthy eyes, and in 100% of the NPDR eyes. Post-suction change in IOP had no statistically significant differences between the two groups (*P* = 0.243).

Tables 2–3 display OCTA measurements after acute elevation in IOP. After the IOP rise in the healthy eyes, vessel density in the whole image of the SCP and DCP, and parafoveal DCP was statistically significantly lower as compared to the baseline (all *P* < 0.05). In addition, the inside disc vessel density was significantly lower as compared to baseline measurements (*P* = 0.017).

In the NPDR group, foveal vessel density at DCP, inside disc vessel density, and peripapillary RNFL thickness decreased significantly after IOP rise (*P* = 0.003, 0.007, and 0.005, respectively) [Table 3].

Comparison of the changes after acute IOP rise between the two groups is presented in Table 4. Considering the differences between the mean ages between the two groups, the comparison was statistically adjusted for the age. None of the measurement changes showed statistically significant differences between the two groups after adjusting for baseline age, except for the peripapillary RNFL thickness (*P* = 0.011).

##  DISCUSSION

In this study, we evaluated the changes in macular and optic disc microvasculature after acute IOP elevation and compared the differences in the eyes of patients without diabetes versus the eyes of diabetics with NPDR. Statistically significant changes in the SCP, DCP, and the inside disc vascular density were observed after acute rise of IOP in the healthy eyes. In the diabetic eyes, however, inside disc vessel density and foveal vessel density at DCP were the only OCTA parameters that changed significantly after IOP elevation. Peripapillary RNFL thickness also decreased significantly in patients with diabetic retinopathy following the IOP rise.

Our findings suggest that the optic disc perfusion as shown by the inside disc vessel density is impaired after an acute IOP elevation of approximately 13 mmHg in healthy and diabetic eyes. However, peripapillary vessel density did not change significantly after an IOP rise. The microvascular perfusion inside the optic disc originates mainly from the posterior ciliary artery, while the central retinal artery supplies the nerve fiber layer.^[27]^ This shows that the microvascular perfusion originating from choriocapillaris may be more susceptible to damage after acute IOP elevation than the occurrence of retinal microvasculature. We observed a decrease in macular perfusion after an IOP rise that was more prominent in the healthy eyes than in the eyes of diabetics. Nagel and Vilser reported a paradoxical response in the size of the veins and arteries after acute elevation of IOP in the healthy eyes.^[30]^ The arterial diameter increased by +1.9% and the venous vessel diameter decreased by –2.6%. Therefore, the net consequence of these changes in the vascular caliber may explain the decrease in vessel density observed in our healthy eyes. The vascular wall in diabetic patients is stiffer, and thus less responsive to the acute rise of IOP.^[31]^ Our study findings correlate with the discoveries of prior studies that suggest that dysfunctional OBF autoregulation may be of importance in the development and progression of glaucoma which may also occur in diabetic individuals.^[18,19]^

Our analyses were also notable for highlighting variations in OCTA indices in eyes with and without diabetic retinopathy before IOP elevation. As expected, at baseline, eyes with NPDR tended to demonstrate lower capillary density and flow in many OCTA indices as compared to eyes without retinopathy.^[23,32]^

Several studies have evaluated the OCTA measurements of the optic disc and/or macula after IOP elevation. Zhang et al^[33]^ did not observe any statistically significant change in optic disc or macular perfusion after moderate IOP elevation (mean IOP rise of 9.6 ± 4.2 mmHg) induced by the dark room prone provocative test (DRPPT) in 40 eyes who were suspected of acute primary angle closure, suggesting efficacy of retinal blood flow autoregulation in response to moderate IOP rise. However, only individuals suspected of angle closure who experienced IOP rise in response to DRPPT were enrolled in this study, thus their results may not be generalizable to other subjects. Similarly, Ma and colleagues^[34]^ found no significant change in either the macular or papillary region perfusion following laser peripheral iridectomy (LPI) in subjects with a 10–20 mmHg IOP rise. However, in people with IOP elevation > 20 mmHg, optic disc and macular perfusion decreased significantly, suggesting a threshold at which autoregulatory mechanisms fail to maintain retinal blood flow. Their study was limited to a subgroup of subjects with occludable angles. In addition, LPI might have adversely affected the image quality and therefore subsequent vessel density measurements.^[35]^ Wen et al^[36]^ investigated optic disc perfusion immediately after the administration of intravitreal anti-VEGF injections and found a significant reduction in optic disc vessel density. Similarly, in a study by Barsash et al^[37]^, both macular and papillary vessel densities decreased following the administration of intravitreal anti-VEGF injections. The superficial macular perfusion was affected more severely as compared to the other regions. In both of these studies, the occurrence of altered image quality^[35]^ after intravitreal injections and including patients with different macular pathology (diabetic macular edema or age-related macular degeneration) were the major shortcomings. In contrast to previously mentioned studies, our study had the advantage of enrolling normal subjects as well as diabetic patients with NPDR.

This study has several limitations. The sample size was small, and Icare rebound tonometry was used instead of applanation tonometry which is considered gold standard for IOP measurements. However, this limitation may have been mitigated as the same standardized method was used to measure IOP in all eyes both before and after IOP elevation, as comparisons of IOP between eyes were all drawn from measurements by the same device. In addition, IOP elevation may not be the same after repetition of the suction procedure. Higher levels of IOP changes may affect the microvascular network differently. We also performed a single IOP measurement after applying the suction and did not check the post-suction IOP to determine probable IOP changes. It is recommended that the clinical significance of the observed changes in microvasculature and retinal structure be explored further. Future analyses with larger sample sizes are warranted.

In summary, the results of our study show that retinal microvasculature in diabetics and healthy eyes respond differently to the acute elevation of IOP. This study provides preliminary insight into changes in OCTA-measured indices after acute IOP elevation, as well as into the potential disturbances in autoregulation that may occur in eyes with and without diabetic retinopathy. As glaucoma and diabetes are highly prevalent causes of vision loss worldwide and are commonly comorbid in patients, a fundamental understanding of potential pathophysiologic similarities is essential. Whether these findings may be applicable to chronic elevation of IOP, as is experienced in the majority of subtypes of glaucoma, remains to be determined.

##  Financial Support and Sponsorship

None.

##  Conflicts of Interest

None declared.
